# Cyclized Peptide Inhibitors of the Small G Protein Cdc42 Mimic Binding of Effector Proteins

**DOI:** 10.1021/acs.biochem.5c00616

**Published:** 2026-01-21

**Authors:** Natasha P. Murphy, George J. N. Tetley, Jefferson Revell, Helen R. Mott, Darerca Owen

**Affiliations:** † Department of Biochemistry, 2152 University of Cambridge, 80 Tennis Court Road, Cambridge CB2 1GA, U.K.; ‡ 4625 AstraZeneca, BioPharmaceutical R&D, Discovery Sciences, Cambridge Biomedical Campus, 1 Francis Crick Ave, Cambridge CB2 0AA, U.K.

## Abstract

The Ras superfamily of small GTPases are challenging targets for therapeutic inhibition, partially due to a lack of pockets amenable to small molecule inhibition. Our previous work identified high-affinity cyclized peptide binders of Cdc42, a member of the Rho family of small GTPases, capable of inhibiting activity. To further optimize these Cdc42 inhibitors, we have engineered modifications to the best sequence available from the original maturation and screened the ability of these third-generation peptides to compete with Cdc42-effector interactions. Improvements in affinity were achieved by single amino acid substitutions at several residue positions. We present the structure of one of these nanomolar affinity, cyclized peptides in complex with Cdc42. The structure reveals that the peptide binds in a β-hairpin conformation to create an extension of the β-sheet of the GTPase Rossman fold, acting as a structural mimic of native Cdc42 effectors. We additionally elucidate the NMR structures of four unbound C-terminal alanine variants and employ both the bound and unbound structures to inform the rational design of substituted peptide inhibitors. Overall, this study expands our understanding of how Ras GTPases can be targeted, by demonstrating a rare example of an inhibitor binding contiguously with a surface of β-strand of the small G protein, which illustrates an innovative avenue for noncovalent therapeutic design.

## Graphical abstract

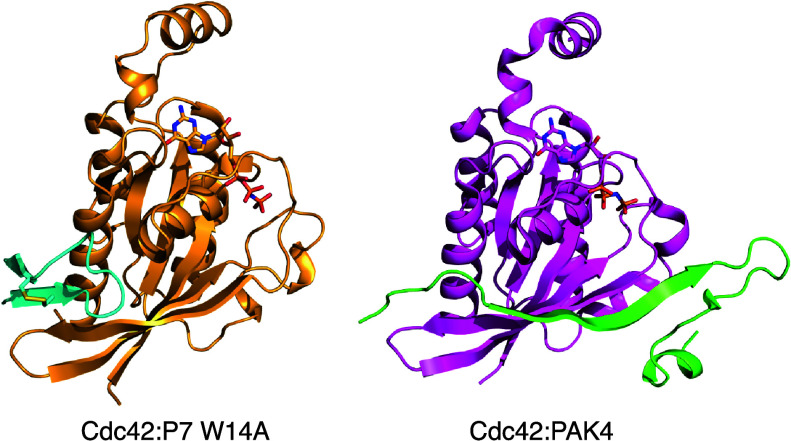

## Introduction

Significant progress has been made in therapeutically targeting the small G protein Ras, the most frequently mutated oncogene in human cancer.^1,2^ Covalent inhibitors have been clinically approved that lock the oncogenic variant of K-Ras, G12C, in its GDP-bound, inactive state by preventing nucleotide exchange.^3,4^ Noncovalent targeting strategies are also being developed for those Ras variants that do not feature nucleophiles amenable to covalent targeting.^5–11^ Despite these milestones, the mechanisms of resistance to the therapeutic inhibition of Ras already observed with other inhibition strategies, including upregulation of the epidermal growth factor receptor,^12^ upregulation of PI3K/AKT/mTOR,^13^ and Aurora kinase A signaling,^14^ and epithelial-to-mesenchymal transition (EMT)^15^ have all been reported in response to Ras G12C covalent inhibitors.

Concurrent with the progress to directly target oncogenic isoforms of Ras, are efforts focused on therapeutic targeting of the wider Ras superfamily members, including the Ral^16–18^ and Rho family GTPases.^19–22^ Within the Rho family there has been particular emphasis on targeting Cdc42 and Rac1, as both are key regulators of the actin cytoskeleton and have been linked to cell invasion and metastasis in several cancers.^23^ Interestingly, some pathways associated with K-Ras inhibitor resistance mechanisms, including EGFR signaling,^24,25^ Aurora kinase A signaling^26^ and activation of the MAPK pathway^27,28^ have been linked to Cdc42, suggesting that its inhibition could be a useful means of counteracting this acquired resistance. Cdc42 itself is not frequently mutated in cancer but alterations are often found in its regulators and downstream effectors, potentially making these further targets for therapeutic intervention.^23^

Most strategies to inhibit Cdc42 employ small molecules (reviewed in^20^). While the cellular and *in vivo* efficacy of small molecule inhibitors targeting Cdc42 has been well documented and include inhibition of tumor growth and angiogenesis,^29^ experimentally determined structures of therapeutics bound to the GTPase and their modes of action are currently lacking.^20^ In contrast, the structures of many Cdc42-effector complexes have been solved^30,31^ and show that most bind via formation of an antiparallel β-sheet between a β-strand in the effector and the β2-strand of the GTPase. Cdc42-effector complexes represent auspicious, but challenging, therapeutic targets. We have previously published an approach to target Cdc42 by inhibiting Cdc42-effector interfaces using peptide inhibitors.^19^ Our second-generation cyclic peptide, P7, bound to Cdc42 with a *K*_d_ of 24 nM. On addition of a cell penetrating sequence, P7 entered A549 cells (human lung epithelial cells, which are homozygous for K-Ras G12S and therefore a good representative of a Ras-driven cancer cell model) and inhibited proliferation in a dose dependent manner. P7 also inhibited migration of A549 cells in Boyden Chamber assays and inhibited both proliferation and migration in wound healing assays. A data-driven docking model of Cdc42 with this peptide suggested that its binding site was partially overlapping with that of a variety of Cdc42 effector proteins. These previous data indicated that P7 is a high affinity Cdc42 binding peptide, possessing many of the properties that would be required in an early lead molecule targeting Ras-driven cancers.

Here we have applied both naïve and rational design strategies to engineer improved affinity into the peptide inhibitor, P7, using natural and noncanonical substitutions to probe an expanded sequence space. We have solved the solution structure of a third-generation peptide bound to Cdc42, which shows that the peptide adopts a β-hairpin conformation and forms a short β-sheet with the β2-strand of Cdc42. The structure demonstrates that, although the peptide was originally derived from a *de novo* selection using a naïve library of random sequences, the peptides mimic the Cdc42 effectors found in nature, binding via an extension of a β-sheet in the GTPase Rossmann fold. Unbound structures adopted by a selection of the modified peptides were also solved and indicate that the β-hairpin conformation is only selected upon binding the small GTPase. Our data reinforces the concept that GTPase-effector interfaces can be selectively targeted by artificially mimicking their natural interactions. As such, these peptide ligands represent promising probes for therapeutically targeting Cdc42.

## Materials and Methods

### Peptide Synthesis of Alanine Scanning Peptide Libraries

Automated peptide synthesis was carried out using a Prelude Protein Technologies peptide synthesizer (Gyros). Fmoc-protected RinkAmide MBHA resin 100–200 mesh (Novabiochem) (0.1 mmol scale) was incubated with each Fmoc-protected amino acid, followed by five wash steps with dimethylformamide (DMF) and dichloromethane (DCM). Deprotections were carried out by two incubations with 40 mL 20% piperidine in DMF. Amino acid couplings were carried out using one solution comprising an activator solution (16% w/v O-(1*H*-6-chlorobenzotriazole-1-yl)-1,1,3,3-tetramethyluronium hexafluorophosphate (HCTU) in DMF), 0.3 M amino acid solutions in DMF and 0.4 M base (*N*,*N*-diisopropylethylamine (DIPEA) in DMF). All residues were double-coupled except for the homocysteine residue (single coupling) and the residues in position 1, 2, and 3 of each sequence (triple coupling). After each amino acid coupling, a capping step was applied using a capping solution (70 mL pyridine, 40 mL acetic anhydride in DMF), followed by three DMF wash steps to remove the capping solution.

After all amino acid couplings were complete, a final manual deprotection step used 40 mL 20% piperidine in DMF for 15 min, before a minimum of three washes of the resins (alternating between 30 mL DCM and 30 mL DMF beginning and ending with DCM), prior to a final drying of the resins using a minimum of four 50 mL diethyl ether washes. The dried resins were cleaved with a cleavage cocktail of 94% trifluoroacetic acid (TFA):3% triisopropylsilane (TIPS):1.5% ethane-1,2-dithiol (EDT):1.5% H_2_O:1.5% thioanisole (ThioA) for a minimum of 5 h. The TFA was removed via rotary evaporation, the crude product precipitated in 40 mL of diethyl ether and the final precipitate retrieved by centrifugation for 5 min at 12,000 g. The ether phase was decanted, crude peptides washed a minimum of four times with 45 mL diethyl ether to remove trace TFA and dried overnight at room temperature before purification by reverse-phase high-performance liquid chromatography (HPLC) purification and cyclization followed by liquid chromatography–mass spectrometry (LC–MS) and analytical HPLC analysis.

### Automated Microwave Synthesis of Interface and Structural Peptide Variant Libraries

Automated peptide synthesis was carried out using a CEM Liberty Blue microwave peptide synthesizer using a CEM Rink-Amide ProTide Resin (0.1 mmol scale) incubated with each Fmoc-protected amino acid. Deprotection and double coupling steps were performed at 90 °C for 4 min for all amino acids, except proline, cysteine and histidine double couplings which were performed for 10 min at 50 °C. Deprotections were carried out using a solution of 20% piperidine in DMF and 0.5 M hydroxybenzotriazole for 15 min. Amino acid couplings were carried out using a solution of 1 M *N*,*N*′-diisopropylcarbodiimide, 1 M 1-hydroxybenzotriazole/ethyl 2-cyano-2-(hydroxyimino)­acetate (Oxyma) and 0.2 M amino acid in DMF.

Manual single couplings of noncanonical residues in the sequences were performed with 4 molar equivalents of the noncanonical amino acid, 4 equivalents of HATU and 8 equivalents of DIPEA with respect to the resin (0.1 mmol scale) for a minimum of 1 h, followed by a minimum of three wash steps (alternating between 30 mL DCM and 30 mL DMF, beginning and ending with DCM). A final deprotection step was employed for resins using a solution of 20% piperidine in DMF with 0.5 M hydroxybenzotriazole for 15 min, before a minimum of three wash steps alternating of 30 mL DCM and DMF, followed by final drying of the resin using a minimum of four 50 mL washes of diethyl ether. The dried resins were cleaved, dried and analyzed as in the previous section.

### Peptide Disulfide Cyclization

Cyclization was carried out using purified linear peptide dissolved in 0.1–0.5 mL acetic acid. Excess acidified iodine in methanol was added dropwise to peptide solutions with stirring until the reaction reached completion, as indicated by no further color change. Any remaining excess iodine was quenched by dropwise addition of 0.5 M ascorbic acid. The final product was purified by reverse phase HPLC and >95% purity verified by LC–MS.

### Reverse-Phase HPLC Purification

Linear and oxidized peptides were purified on a reverse-phase preparative HPLC (Waters xBridge 5 μm, 19 × 250 mm C18 OBD column) linked to a Varian or Waters 2767 analytical HPLC system at a flow rate of 20 mL/min, with detection by UV absorption at 214 nm.

### LC–MS and Analytical HPLC Analysis

Peptide samples were loaded on to an Agilent Polaris C8-A column (4.6 × 100 mm, 3 mm) coupled to a UV detector at 210 nm and coupled to a Waters 3100 mass detector (ESI+ mode) and eluted with a gradient of acetonitrile (with 0.1% v/v TFA). For analytical HPLC analysis, samples were eluted with 10–90% MeCN (0.1% v/v TFA) in water (0.1% v/v TFA) over 15 min at 1.5 mL min^–1^ at 40 °C with detection at 210 nm on an Agilent Polaris C8-A column (4.6 × 100 mm, 3 mm).

### Recombinant Peptide Synthesis

DNA encoding the sequence PSICHVHRPDWPCAYR was cloned into pET-31b­(+) (Merck) using overlapping oligonucleotides (Sigma-Aldrich) and expressed in inclusion bodies in *E*.*coli* BL21 (DE3) cells. Uniform ^15^N-labeling was achieved using ^15^N-ammonium chloride in M9 minimal growth media supplemented with 0.4% (w/v) glucose. Cultures were induced with 1 mM IPTG at A_600_ of 0.8 and incubated at 37 °C with shaking for 5 h. After harvest, cells were lysed in 50 mM Tris-HCl pH 7.5, 150 mM NaCl, 10 mM DTT, 1% Triton buffer, supplemented with 1 mM PMSF and EDTA-free protease inhibitor cocktail (Sigma-Aldrich), by four passes through an Emulsiflex C5 (Avestin). The lysate was centrifuged for 20 min, at 12,000 g, 4 °C. The pellet was washed using a series of resuspension and centrifugation steps using 50 mM Tris-HCl pH 7.5, 150 mM NaCl, 10 mM DTT, 1% Triton buffer and 50 mM Tris-HCl pH 7.5, 1 M NaCl, 10 mM DTT, 2% Triton and finally resuspended in 6 M guanidine-HCl pH 8.0, 10 mM DTT.

Dropwise addition of 1 M HCl to the resuspended inclusion bodies was carried out until a pH of 1 was achieved, before incubation at 85 °C for 5 h. The resulting cleaved peptide was dialyzed against 5 L of 50 mM sodium acetate pH 3.6, 10 mM DTT, overnight at 4 °C. The dialyzed peptide was spun at 20,000 g, 4 °C to remove debris and the supernatant was purified using a HiTrap S HQ column (Cytiva) in 50 mM sodium acetate pH 3.6, 10 mM DTT and a salt gradient of 0–1 M NaCl. Fractions containing the peptide were pooled and lyophilized, dissolved in water and oxidized by an iodine oxidation. Excess acidified iodine (in methanol) was added dropwise to peptide solutions with stirring until the reaction reached completion, as indicated by no further color change. Any remaining excess iodine was quenched by dropwise addition of 0.5 M ascorbic acid. Postoxidation the peptide was purified by reverse-phase HPLC purification on a XBridge Prep C18 10 × 50 mm column using a gradient of 5–50% acetonitrile in water. The correct mass of purified oxidized peptide was confirmed by LC/MS and lyophilized.

### Protein Expression and Purification

Proteins were expressed from pGEX vectors (Cytiva) as GST fusion proteins. Cdc42 (UniProtKB P60953) Q61L Δ7 expression was carried out in *E*. *coli* BL21 grown in 2TY media (for unlabeled samples) or M9 minimal media supplemented with ^15^NH_4_Cl (for uniformly ^15^N-labeled protein) or MOPS media supplemented with both ^15^NH_4_Cl, ^13^C-glucose and 5% ^15^N, ^13^C labeled Celtone (Cambridge Isotope Laboratories Inc.) (for double-labeled protein). Cells were grown to an A_600_ of 0.8 at 37 °C before induction with 0.1 mM isopropyl-β-D-thiogalactopyranoside (IPTG) for 6 h at 37 °C. Cells were harvested and lysed in 50 mM Tris-HCl pH 7.5, 150 mM NaCl, 5 mM MgCl_2_, 5 mM DTT supplemented with EDTA-free protease inhibitor cocktail (Merck) and 1 mM PMSF. Cdc42 was purified using Glutathione-agarose (Merck) and cleaved using restriction-grade Thrombin protease (Merck), before purification using size exclusion chromatography (S75 16/600, Cytiva) in 50 mM Tris-HCl pH 7.5, 150 mM NaCl, 5 mM MgCl_2_, 5 mM DTT. Cdc42 proteins were exchanged with [^3^H]­GTP or GMPPNP as described previously.^32^

### Scintillation Proximity Assays

Affinities of Cdc42 for the GST-tagged WASP (UniProtKB P42768), ACK (UniProtKB Q07912) or PAK1 (UniProtKB Q13153) GBDs were measured using direct SPAs as previously described.^33^ GST-Cdc42 effector (30 nM GST-ACK GBD, 20 nM GST-PAK1 GBD or 5 nM GST-WASP GBD) were immobilized on Protein SPA fluoromicrosphere via an anti-GST antibody (Sigma-Aldrich) and the SPA signal monitored in the presence of varying concentrations of [^3^H]­GTP·Cdc42. Binding curves were fitted using a direct binding isotherm to obtain *K*_d_ values and their standard errors. For competition assays, [^3^H]­GTP·Cdc42 and GST-effector GBD were added to beads at a concentration equivalent to the *K*_d_ determined by direct binding and the SPA signal monitored in the presence of varying concentrations of peptide inhibitor. The data were fitted by nonlinear regression to a one-site competitive binding isotherm in the Graphit5 software package (Erithacus Software Ltd.) to obtain *K*_d_ values and standard errors.

### NMR Experiments

NMR spectra were recorded on Bruker Avance III AV800 and AV600 spectrometers at 298 K. 2D TOCSY (65 ms mixing time) and NOESY (250 ms mixing time) were recorded on peptide samples prepared at ∼2 mM in 50 mM sodium phosphate buffer pH 7.0 with 10% (v/v) D_2_O. The following complex samples were prepared: ^13^C, ^15^N-labeled Cdc42 with unlabeled W14A peptide, ^15^N-labeled Cdc42 with unlabeled W14A peptide and unlabeled Cdc42 with ^15^N-labeled W14A peptide using a 50 mM sodium phosphate pH 6.8, 150 mM NaCl, 5 mM MgCl_2_, 0.1 mM GMPPNP, 10% (v/v) D_2_O buffer. Final complex samples were prepared with 10–20% excess of the unlabeled component.

^15^N HSQC, ^15^N NOESY-HSQC, ^15^N TOCSY-HSQC were recorded on ^15^N-labeled Cdc42·GMPPNP with unlabeled P7 W14A. ^13^C-separated NOESY, HCCH TOCSY, HNCA, HNCACB, HNCOCA, HNCOCACB, ^13^C-HSQC, ^13^C-CT-HSQC, ^13^C-filtered, ^13^C-separated NOESY and TROSY were recorded on ^13^C,^15^N-labeled Cdc42·GMPPNP with unlabeled P7 W14A. The P7 W14A resonances in the complex were assigned using 3D ^15^N-separated NOESY and ^15^N-separated TOCSY experiments recorded on ^15^N-labeled P7 W14A with unlabeled Cdc42·GMPPNP.

All NMR data were processed using the AZARA package (W. Boucher https://www.cambridge2000.com/azara/) and analyzed using CCPN Analysis software version 2.4.^34^

### Structure Calculations

Distance restraints were exported from CCPN analysis V2.4. All structures were calculated using ARIA 1.2 ^35^ interfaced to CNS, where structures were calculated iteratively for 8 iterations in total, with a final ensemble of structures of the 35 lowest energy structures calculated.

Dihedral angles for Cdc42 were generated using TALOS-N.^36^ Hydrogen bond restraints were included to reinforce the secondary structure of Cdc42. After the first rounds of structure calculation hydrogen bond restraints were added within P7 W14 and between the protein and peptide, where their presence could be inferred by the NOE patterns.

## Results and Discussion

Previously we subjected a 16-mer disulfide-linked peptide to a CIS-display maturation selection to generate a panel of peptides with increased affinity for Cdc42. One of the highest affinity peptides (P7), which also showed improved specificity for Rac1 and Cdc42 over other Rho family GTPases and K-Ras, was characterized structurally and functionally.^19^ The P7 peptide was the starting point in this study, for both naïve and rational design peptide engineering programmes, to further improve affinity and introduce properties important for therapeutic peptides.

### Alanine Scan of P7

To provide residue-specific insights into which side chains of P7 (PSICHVHRPDWPCWYR) were required for binding Cdc42, an alanine scan was undertaken. Fourteen peptides were synthesized (the disulfide bonded Cys4 and Cys13 were left unchanged), each with a single residue changed to Ala, and their affinity for Cdc42 measured (Figure 1, Table 1).

**Figure 1:**
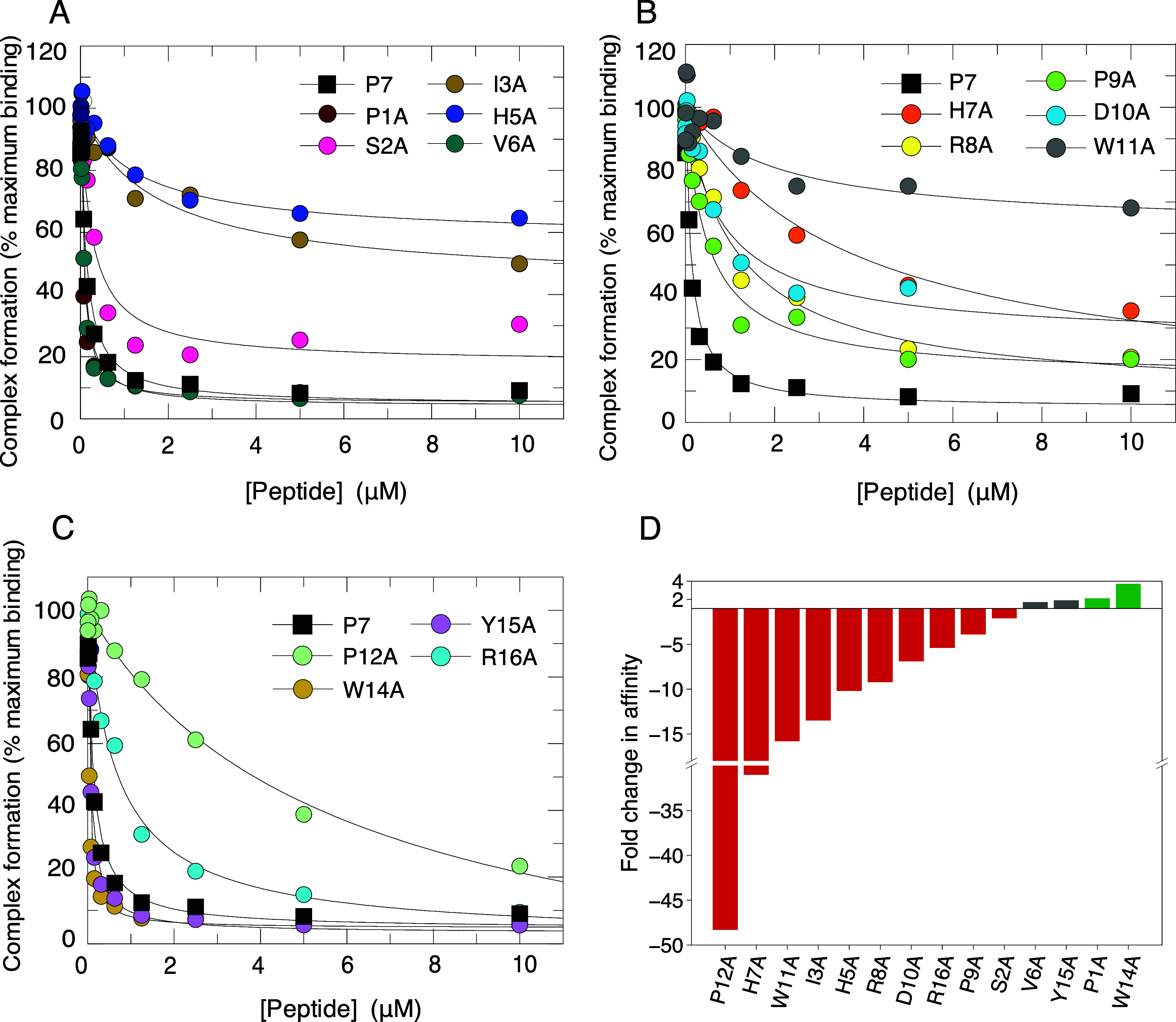
Affinity of P7 alanine scan peptides for Cdc42 measured by competition SPA. A–C. Displacement of [^3^H]­GTP·Cdc42 from GST-WASP by P7 and its variants. Increasing concentrations of each peptide were titrated into 5 mM [3H]­GTP·Cdc42 and GST-WASP 210–321. The *K*_d_ value for Cdc42 binding to GST-WASP 210–321 was fixed to the value (5 nM) obtained in direct SPAs. The data were fitted to an isotherm describing a full competition model^33^ to calculate the *K*_d_ values. The data and curve fits are displayed as a percentage of the maximal SPA signal, shown combined for repeats and the numerical *K*_d_ values and associated errors are presented in Table 1. The control P7 peptide is shown as filled-black squares on each graph. D. Bar chart displaying the average fold change in affinity of each variant relative to the control. Green bars represent variants which bound with a greater than 2-fold affinity improvement relative to P7, gray bars, variants which bound with an affinity within ±2-fold of the control (which were considered not significant), and red bars, variants which bound with at least a 2-fold decreased in affinity compared to P7.

**Table 1:** Affinities of Alanine Scan P7 Variants for Cdc42

**Variant**	***K***_**d**_ ^a^ **(nM)**	**Δ*G*** (cal/mol)	**ΔΔ*G*** (cal/mol)	**Fold change in affinity**
**P7** (PSICHVHRPDWPCWYR)	52 ± 13	–9765		
**P1A** (**A**SICHVHRPDWPCWYR)	25 ± 9	–10191	–426	2.1 ↑
**S2A** (P**A**ICHVHRPDWPCWYR)	107 ± 35	–9344	420	2.1 ↓
**I3A** (PS**A**CHVHRPDWPCWYR)	704 ± 279	–8248	1517	13.5 ↓
**H5A** (PSIC**A**VHRPDWPCWYR)	529 ± 224	–8414	1351	10.2 ↓
**V6A** (PSICH**A**HRPDWPCWYR)	30 ± 7	–10085	–320	1.7 ↑
**H7A** (PSICHV**A**RPDWPCWYR)	1612 ± 516	–7765	1999	31.0 ↓
**R8A** (PSICHVH**A**PDWPCWYR)	479 ± 93	–8472	1293	9.2 ↓
**P9A** (PSICHVHR**A**DWPCWYR)	202 ± 39	–8974	790	3.9 ↓
**D10A** (PSICHVHRP**A**WPCWYR)	357 ± 146	–8643	1122	6.9 ↓
**W11A** (PSICHVHRPD**A**PCWYR)	824 ± 880	–8156	1609	15.8 ↓
**P12A** (PSICHVHRPDW**A**CWYR)	2512 ± 722	–7507	2257	48.3 ↓
**W14A** (PSICHVHRPDWPC**A**YR)	14 ± 4	–10528	–764	3.7 ↑
**Y15A** (PSICHVHRPDWPCW**A**R)	27 ± 6	–10146	–382	1.9 ↑
**R16A** (PSICHVHRPDWPCWY**A**)	283 ± 53	–8778	986	5.4 ↓

a Affinities calculated from competition SPAs against a Cdc42-WASP complex. Average *K*_d_ values are quoted with errors from curve fitting.

Most variants displayed a reduced affinity (>2-fold) relative to P7, with the most detrimental alanine substitutions at Ile3, His5, His7, Trp11 and Pro12 (all >10-fold decrease). Four variants bound with higher affinity than P7, but only P1A and W14A (hereafter referred to as P7 W14A) showed increases greater than 2-fold. These data showed the contribution of individual amino acids to the binding energy of the complex and therefore where improvements could potentially be targeted.

### NMR Analysis of Free P7 W14A and the Cdc42·GMPPNP-P7 W14A Complex

To probe the impact of the substitutions in the P7 variants, P7 W14A was selected for structural analysis as it had the largest increase in affinity for Cdc42. In the initial 2D-NOESY and TOCSY NMR spectra recorded on the parent peptide, free P7, three peaks were observed for Trp Hε, which were readily identified by their distinctive chemical shifts, although the sequence contained only two Trp residues. This indicated that there were two species present, whose exchange was occurring on a slow time scale, permitting both to be observed. To rule out the possibility that this was due to disulfide bond instability, excess reducing agent was added but this had no effect (data not shown). We reasoned that one of the three proline residues in the peptide was undergoing *cis–trans* isomerization and affecting one of the tryptophan residues. *Cis–trans* isomerization has been observed within the intercysteine loop of disulfide-containing peptides and is increasingly recognized as a structural feature unique to peptide conformation.^37,38^ Inspection of the peptide sequences suggested that Pro12 was the most likely candidate as Trp-Pro sequences are known to favor a *cis*-peptide bond (here between Trp11 and Pro12).^39^ Experiments recorded on the free P7 W14A peptide, which only contains a single Trp, showed two peaks for Trp11 HεNε. We determined the structure of this peptide and, based on the pattern of NOEs observed, the major species in P7 W14A was identified as the Trp11-Pro12 *trans* isomer. Like the original P7 peptide,^19^ P7 W14A does not form any regular secondary structure (Figure 2A, Table S1), but forms a loose hairpin, braced by the disulfide bond. The hairpin turn is stabilized by a hydrogen bond between Arg8 and Trp11, with Pro9 and Asp10 at the apex of the turn.

**Figure 2:**
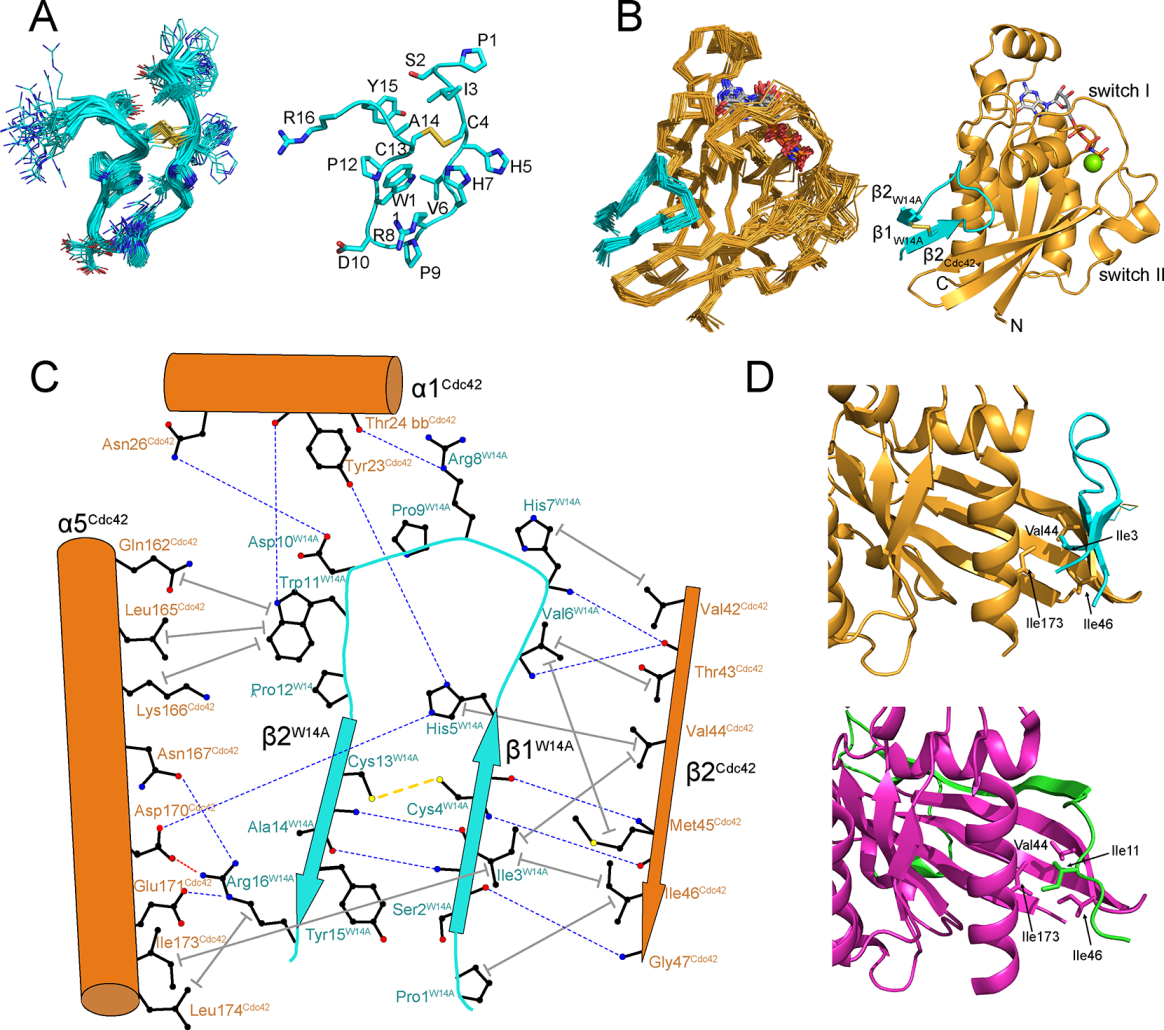
Structures of P7 W14A and the Cdc42:P7 W14A complex. A. Structure of free P7 W14A peptide (PDB 9grk). The family of structures is shown on the left with a cartoon backbone and the side chains in wire frame. The closest structure to the mean is shown on the right. Atoms are colored as follows: nitrogen - dark blue, oxygen - red, sulfur - yellow, carbon - cyan. B. Structure of the Cdc42:P7 W14A complex (PDB 9grm). The family of structures is shown on the left and the closest structure to the mean is on the right. Cdc42 is shown in orange and P7 W14A is cyan. The nucleotide and disulfide bond are shown in a stick representation, with carbon colored gray, nitrogen blue, oxygen red, sulfur yellow and phosphorus orange. The Mg^2+^ ion is shown as a green sphere. The newly formed β-strands in the peptide, which extend the β-sheet from β2 of Cdc42, are indicated. C. Schematic of contacts between Cdc42 (orange) and P7 W14A (cyan). All the peptide side chains, Cdc42 side chains that make substantial contacts and main chain nitrogen and oxygen involved in hydrogen bonds are shown in a ball and stick representation. Carbon is shown in black, nitrogen in blue and oxygen in red. The salt bridge is shown as a red dashed line, hydrogen bonds are shown as blue dashed lines and the peptide disulfide bond is a yellow dashed line, with lines joining the relevant atoms. Nonpolar contacts are indicated by a thick gray solid line between the side chains with a bar at each end to indicate that multiple atoms are involved. The secondary structural elements are shown as arrows and cylinders for β-sheets and α-helices, respectively. D. Comparison of P7 W14A and PAK4 binding to Cdc42 (PDB 5upk). Top: closeup of the Cdc42 (orange): W14A P7 (cyan) complex. Bottom: the same view of the Cdc42 (pink): PAK4 (green) complex. The conserved Ile side chain from the CRIB effectors (Ile11 in PAK4) binds in a hydrophobic pocket formed by Val44, Ile46 and Ile173 from Cdc42 and this is mimicked by Ile3 in the P7 W14A peptide.

Previously we generated a data-driven model of the Cdc42-P7 complex,^19^ however the peptide and several regions of Cdc42 are likely to sample different conformations in their unbound states. This plasticity indicates that the structures could change upon binding, representing a challenge for molecular docking. To probe this plasticity, we therefore employed a combination of 3D and 2D NMR experiments to determine an experimentally derived structure for P7 W14A in complex with Cdc42.

^15^N HSQC experiments recorded on P7 W14A indicated the presence of two species visible for several residues around Trp11, as expected (Figure S1). Addition of excess unlabeled Cdc42·GMPPNP led to observation of a single species, indicating conformational selection of one isomer by the binding partner. The chemical shift changes when P7 W14A was titrated into ^15^N-labeled Cdc42·GMPPNP showed that the nucleotide-sensitive switch regions of the G protein were unlikely to be involved in the interaction (data not shown), this is consistent with the results obtained when P7 was titrated into Cdc42·GMPPNP.^19^ The switch regions of Rho family GTPases are involved in conformational dynamics on a time scale that renders them invisible in NMR experiments but when they are fixed by binding to other proteins, their NMR signals reappear.^19^ In the P7 W14A complex, signals for the switch regions were still absent, reinforcing their lack of interaction.

NOESY experiments recorded on the complex were used to derive 3,868 distance restraints, of which there were 3,302 unambiguous and 556 ambiguous in the final iteration. Of these, there were a total of 128 intermolecular distance restraints (Table S2). These were subsequently used to determine the structure of the Cdc42-P7 W14A complex.

### The Structure of Cdc42-P7 W14A Reveals a Peptide Transition to a β-Hairpin

The structure of the P7 W14A-Cdc42 complex reveals that the W14A peptide adopts an ordered β-hairpin in the complex (Figure 2B). The β-strands, comprising residues 2–5 and 13–15, extend the β-sheet in the G protein. The first β-strand of W14A runs antiparallel to β2 of Cdc42, with its position fixed by distance restraints between the amides of Met45^Cdc42^ and Gly47^Cdc42^, and the amides of Cys4^W14A^ and Ser2^W14A^ respectively (Figure S2A), as well as to the side chains of Cys4^W14A^ and Val6^W14A^. These main chain interactions were reinforced by several peaks in the X-filter experiment between the methyl groups of Thr43^Cdc42^, Val44^Cdc42^, Met45^Cdc42^, Ile46^Cdc42^ and the side chains of residues 3 (Ile) and 6 (Val) in the peptide (Figure S2B). In addition, methyl groups of residues in Cdc42 helix α5 (Leu165, Ile173, Leu174) show NOEs to both sides of the P7 W14A β-hairpin (Figure S3).

Within the P7 W14A peptide, long-range backbone NOEs were observed between the N- and C-termini that stabilize the β-hairpin, for example, Ser2^Hα^-Arg16^NH^, Ile3^NH^-Ala14^NH^ (contacts are summarized in Figure 2C). In the complex, residues Ile3^W14A^ and His5^W14A^ in β1^W14A^ are buried, making contacts with β2^Cdc42^, α1^Cdc42^ and α5^Cdc42^. Ile3^W14A^ forms hydrophobic interactions, whereas the side chain of His5^W14A^ forms hydrogen bonds with Tyr23^Cdc42^ and Asp170^Cdc42^. This arrangement means that the disulfide bond between Cys4^W14A^ and Cys13^W14A^ is on the exterior of the complex (Figure 2B). The P7 W14A β1 strand finishes at His5^W14A^, so that the backbone twists, partially exposing His7 ^W14A^ and peeling the peptide away from β2 ^Cdc42^ and toward α1^Cdc42^. Arg8^W14A^ forms a hydrogen bond with the backbone of Thr24^Cdc42^, which is followed by Pro9^W14A^ at the apex of the turn. Asp10^W14A^ also contacts α1^Cdc42^, hydrogen bonding with Asn26. Trp11^W14A^ is packed into a pocket formed by the side chains of Tyr23^Cdc42^, Gln162^Cdc42^, Leu165^Cdc42^ and Lys166^Cdc42^. The only other residue that makes substantial contacts with Cdc42 is Arg16^W14A^, which forms a hydrogen bond with Asn167^Cdc42^ and a salt bridge with Asp170^Cdc42^. Ala14^W14A^ makes very little contact with Cdc42. In the parent P7 peptide the tryptophan at this position was less favorable for binding. The presence of a larger tryptophan side chain here could result in clashes with Lys166^Cdc42^ and Asp170^Cdc42^.

The experimental structure is very different to the data-driven model of P7 with Cdc42 that we previously generated.^19^ However, that model was derived using sparse data on the peptide side. Furthermore, there are clear, significant differences between the structures of the unbound and bound P7 W14A peptide, indicating a transition from a loose hairpin with no fixed secondary structure to the β-hairpin (Figure 2A and B). This reorientation is driven by the interactions between the side chains of the peptide and Cdc42. Given the conformational changes between the free and bound peptides, as well as the well-documented allostery of small G proteins,^40^ it is not surprising perhaps that the data-driven docking was unsuccessful.

This experimental structure therefore represents the binding mode of this family of peptides. The Cdc42-P7 W14A structure therefore provides a context in which to rationalize the alanine scan of the P7 peptide (Figure 1, Table 1). Ile3, His5, and Trp11 are buried in the complex and each makes multiple contacts with Cdc42. Arg8, Asp10 and Arg16 form hydrogen bonds or salt bridges. If any of these side chains are changed to Ala, the affinity is reduced, with the three buried residues being more deleterious. The P12A and H7A variants have the largest effect in the alanine scan, yet neither are completely buried in the complex. His7 makes some contacts and it is likely that these are necessary to orient the side chains of Arg8 and Asp10 to allow optimum interactions (Figure 2C). On the other hand, Pro12 does not make any substantial contacts with Cdc42, suggesting that its effects must be purely structural. The second β-strand in P7 W14A comprises residues 13–15 with Pro12 (which is opposite His5 in the first β-strand) preventing extension of the second strand toward the N-terminus. Presumably its replacement with Ala would allow the strand to extend, destabilizing the interactions made by Asp10 and Trp11. Pro9 resides at the tip of the hairpin loop and although it does not contact Cdc42, a proline is essential here to facilitate the β-turn.

### Comparison of the Binding Modes of P7 W14A and Native Cdc42 Effectors

Fascinatingly, the structure reveals that the P7 W14A peptide acts as a partial mimic of the CRIB effectors that bind Rac1 and Cdc42. The GTPase binding regions of these effectors utilize an ISxP consensus sequence, just N-terminal to a β-strand that runs antiparallel to Cdc42 β2, followed by a conserved FxHxxH that interacts with Cdc42 switch I. More divergent members of the CRIB family, such as Par6 and IRSp53 form the intermolecular β-sheet but lack the FxHxxH motif. The P7 W14A peptide has neither of these motifs but is reminiscent of the CRIB effectors, in that it extends the β-sheet structure of Cdc42 upon binding, although the region of Cdc42 β2 that is involved is not identical (Figure S4). Comparison with the structure of Cdc42-PAK4 (Figure 2D) shows that Ile3^W14A^ is equivalent to Ile11^PAK4^. In both complexes the isoleucine is buried in a pocket formed by Val44, Ile46 in β2^Cdc42^ and Ile173 in α5^Cdc42^. Therefore, a peptide, originally selected from a naïve library, binds Cdc42 with a high affinity and mimics a natural binding motif. This similarity also explains why the P7 peptides can compete with the CRIB proteins in binding assays, even though it does not interact with the nucleotide-sensitive switch regions. In the CRIB proteins, binding to Cdc42 is stabilized by extensive interactions C-terminal to this isoleucine. In the case of our peptides, the disulfide bond is necessary to stabilize formation of a β-hairpin, which brings a number of side chains into the correct orientation to bind Cdc42 (Figure 2C).

Previously, we established that P7 also binds to active Rac1 but with a 30-fold lower affinity than Cdc42, that K-Ras binding was 1000-fold weaker and that binding to RhoA and RalA were too weak to be estimated.^19^ As Ala14 makes no contacts with Cdc42, most of the interactions are likely to be similar in P7 and P7 W14A. Of the 15 Cdc42 residues whose side chains contact P7 W14A, 11 are conserved in Rac1 (Figure S5), explaining why P7 can bind Rac1. RhoA and representative Ras family members only have 6–8 residues conserved, explaining their lower affinities. An important difference between the CRIB effectors and P7 W14A is that P7 W14A does not engage the switch regions at all (Figure 2B). Unlike the effectors, which bind specifically to Cdc42·GTP (due to the conformation of the switches), peptide P7 had similar affinities for Cdc42 bound to GDP and GMPPNP.^19^ Based on the interactions observed here, we assume that P7 W14A binding to Cdc42 is also nucleotide independent.

### Structure-Informed Rational Design of Peptide Variants

Based on the alanine scan data and our structure of the Cdc42-P7 W14A, we proceeded to design and test two further panels of peptide variants. The first group of variants were designed to directly enhance interface contacts (interface-targeted mutants) and the second to affect the peptide structure itself and its subsequent binding to Cdc42 (structural-targeted mutants). Non-canonical residues were incorporated primarily to increase affinity but also to increase proteolytic stability, an important feature for therapeutic peptides.^41^

#### Interface Variants

Interface variants were designed to modify the side chains of P7 residues that directly interact with Cdc42. The binding surface on Cdc42 comprises three key secondary structure elements (α1, α5 helix and β2), which form a pocket into which the P7 W14A hairpin inserts (Figure 2C). The structure of the free P7 W14A peptide (Figure 2A) showed that the disulfide bond allowed the formation of a loop with Arg8 and Asp10 at its apex and alanine scan data (Figure 1, Table 1) showed that changes to all residues in this loop except Val6 were detrimental to binding. Two of these residues are prolines (Pro9 and Pro12), neither of which makes direct contact with Cdc42, but whose restricted backbones could contribute to the orientation of the loop. Substitutions were therefore targeted at the remaining peptide residues in the loop, at position His5, His7, Arg8, Asp10, and Trp11. In the structure, His5^P7 W14A^ forms a hydrogen bond with Tyr23^Cdc42^: this was changed to Tyr, which is larger but can still form a hydrogen bond. His7^P7 W14A^ lies along the surface of Cdc42, making polar contacts with the backbone of Thr24^Cdc42^ and hydrophobic interactions with the side chain of Val42^Cdc42^. This was changed to Phe and Tyr, to elucidate the contributions of the hydrophobic and hydrogen bond interactions. Arg8^P7 W14A^ forms hydrogen bonds with the backbone of Thr24^Cdc42^ and was changed to Lys as an alternative positively charged residue. Asp10^P7 W14A^ forms a hydrogen bond with Asn26^Cdc42^ and was changed to Glu, to ascertain whether a longer acidic side chain could make more favorable contacts. Finally, Trp11^P7 W14A^ is buried in a hydrophobic pocket comprising Cdc42 residues Tyr23, Gln162, Leu165 and Lys166. This tryptophan was changed to phenylalanine to test whether closer contacts with this smaller side chain could increase affinity. A 5-bromotryptophan (BrW) side chain was also tested at this position, which could potentially form a halogen bond with the side chain of Lys166^Cdc42^.

All the interface variants were synthesized and their binding to Cdc42 measured using competition SPAs (Figure 3A and B, Table 2). Most of the interface variants showed decreased affinity over the parent peptide (P7) but some interesting improvements were identified. The results of substituting His7^P7^ were contrasting; the H7Y peptide showed a 7.5-fold enhancement in affinity, while the H7F variant had a slightly reduced affinity of 1.4-fold relative to P7. The combined data for this pair of substitutions suggest that the hydroxyl group of Tyr7 is necessary for enhanced contact with Cdc42 or that the H7F substitution is not tolerated because phenylalanine is too hydrophobic for this surface position. Small affinity losses of 1.9 and 1.6-fold were seen for the R8K and D10E variants respectively (Figure 3A, Table 2), smaller than the losses when these were replaced with alanine, reflecting the conservative nature of these substitutions. The loss of affinity suggests that the Arg8^P7^-Thr24^Cdc42^ and Asp10^P7^-Asn26^Cdc42^ hydrogen bonds strengths are already maximized with these side chains. The Trp11^P7^ variants also yielded interesting results. The W11BrW modification resulted in a significant enhancement in affinity (9-fold), whereas the W11F mutant decreased affinity by 2.8-fold (Figure 3B, Table 2). This indicates that the smaller phenylalanine ring is less favorable but the effect of the 5-bromotryptophan substitution suggests that the predicted halogen bond improves the affinity significantly.

**Figure 3:**
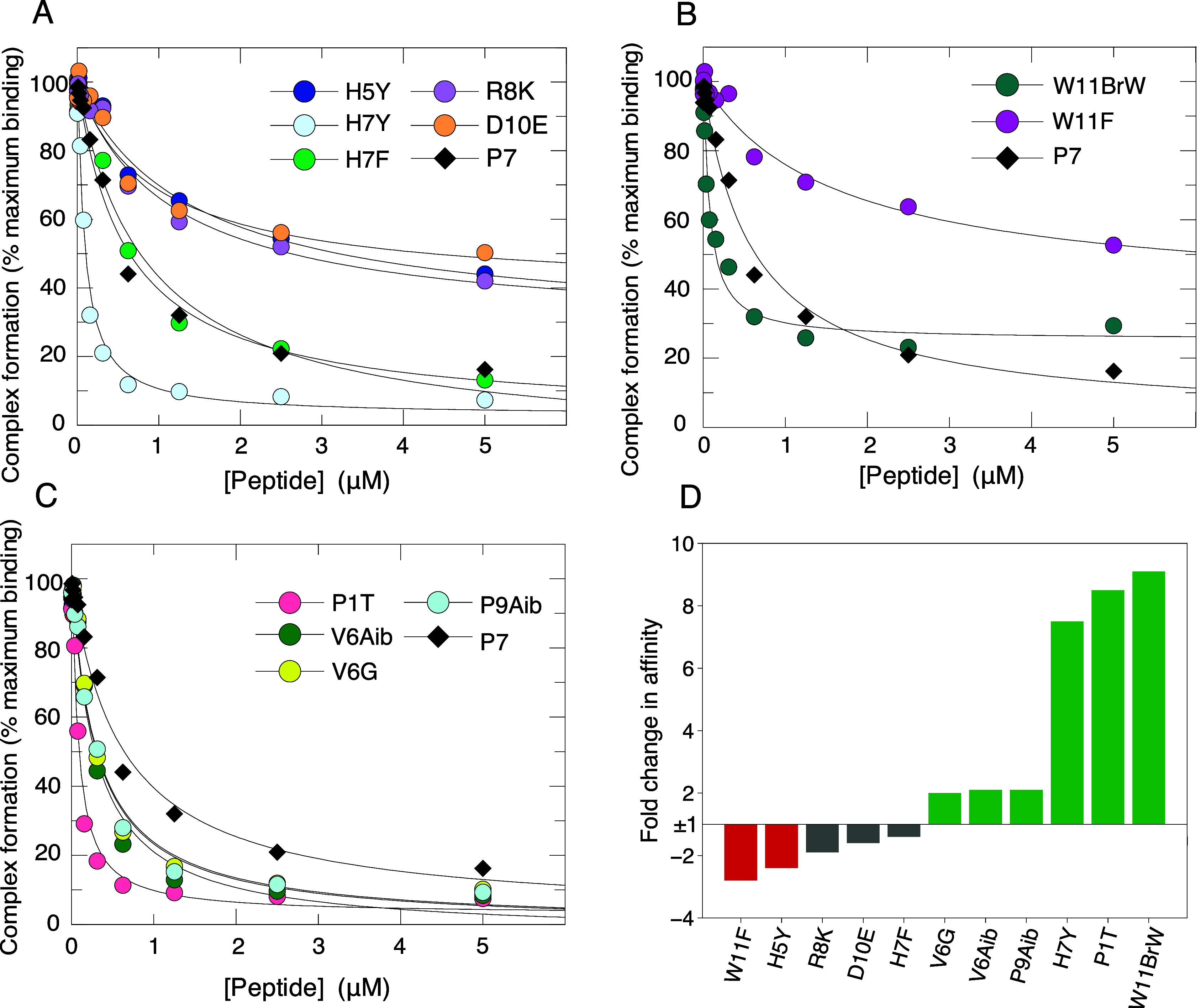
Affinity of Interface and Structural-Targeted P7 Variants for Cdc42. A–C. Displacement of [^3^H]­GTP·Cdc42 from GST-PAK1 by P7 and its variants. Increasing concentrations of each peptide were titrated into 20 mM [3H]­GTP·Cdc42 and GST-PAK1 75–132. The *K*_d_ value for Cdc42 binding to GST-PAK1 75–132 was fixed to the value (20 nM) obtained in direct SPAs. The data were fitted to an isotherm describing a full competition model^33^ to calculate the *K*_d_ values. The data and curve fits are displayed as a percentage of the maximal SPA signal, shown combined for repeats and the numerical *K*_d_ values and associated errors are presented in Table 2. ‘Interface-targeted mutants’ are divided across panels A and B for clarity, with panel C displaying structural-targeted variants. D. Bar chart displaying the fold change in affinity of each variant relative to wildtype, P7. Green bars represent variants which bound with a greater than 2-fold improvement in affinity relative to the P7 control; gray bars, variants which bound with an affinity within ±2-fold of the control (which were considered not significant) and red bars, variants which bound with at least 2-fold decreased affinity relative to the P7 control.

**Table 2:** Affinities of Rationally Designed P7 Variants for Cdc42

**Variant**	**Sequence**	***K***_**d**_ ^a^ **(nM)**	**Fold Change in affinity**
**P7**	PSICHVHRPDWPCWYR	247 ± 48	
**P1T**	**T**SICHVHRPDWPCWYR	29 ± 7	8.5 ↑
**H5Y**	PSIC**Y**VHRPDWPCWYR	587 ± 141	2.4 ↓
**V6Aib** ^b^	PSICH**Aib**HRPDWPCWYR	117 ± 27	2.1 ↑
**V6G**	PSICH**G**HRPDWPCWYR	122 ± 23	2.0 ↑
**H7Y**	PSICHV**Y**RPDWPCWYR	33 ± 8	7.5 ↑
**H7F**	PSICHV**F**RPDWPCWYR	353 ± 93	1.4 ↓
**R8K**	PSICHVH**K**PDWPCWYR	475 ± 136	1.9 ↓
**P9Aib**	PSICHVHR**Aib**DWPCWYR	120 ± 20	2.1 ↑
**D10E**	PSICHVHRP**E**WPCWYR	383 ± 126	1.6 ↓
**W11BrW** ^c^	PSICHVHRPD**BrW**PCWYR	27 ± 7	9.1 ↑
**W11F**	PSICHVHRPD**F**PCWYR	683 ± 237	2.8 ↓

a Affinities calculated from competition SPAs against a Cdc42-PAK complex. Average *K*_d_ values are quoted with errors from curve fitting.

b Denotes 2-aminoisobutyric acid.

c Denotes 5-bromotryptophan.

#### Structural Variants

Val6 had been previously identified as contributing to the small hydrophobic core in the unbound P7 peptide,^19^ however its replacement with Ala increased the affinity slightly (Figure 1A, Table 1), suggesting that peptide compaction could be important. As glycine is smaller than alanine, a V6G variant was also tested. In addition, Val6 was changed to the α-substituted alanine variant 2-aminoisobutyric acid (α-methylalanine, Aib), to introduce proteolytic stability into the peptide. The P7 peptide includes three proline residues, Pro1, Pro9 and Pro12, which would be expected to lend some rigidity to the backbone. The P1A substitution (Figure 1A, Table[Table tbl1]) marginally increased the affinity, suggesting that a Pro at this position is not essential or even desirable. In our previous maturation screen,^19^ a peptide with a threonine at position 1 bound Cdc42 with a high affinity, so this variation was tested in the P7 peptide. Of the remaining Pro residues, replacing Pro12^P7^ with alanine reduced the affinity 48-fold (Figure 1C, Table 1), suggesting that this residue is important for the integrity of the peptide structure in the complex. The effect of the P9A variant was less severe (Figure 1B, Table 1). This residue is in the helical region of the Ramachandran plot in both the free P7 structure (PDB 6r28) and in the P7 W14A-Cdc42 complex, so a P9Aib variant was generated to favor helical backbone angles.

All structural-targeted variants were synthesized and analyzed for binding to Cdc42 by competition SPAs and their affinities compared to the P7 control peptide (Figure 3C, Table 2). All variants displayed some enhanced affinity relative to the P7 control. The greatest enhancement in affinity was achieved by the P1T peptide, which bound Cdc42 8.5-fold tighter than P7. Pro1 does not contact Cdc42 directly but it is possible that a threonine at this position can reorient toward Cdc42 and make some favorable contacts. Both the V6Aib and V6G mutants displayed a modest, 2-fold, improvement in affinity relative to P7 (similar to the V6A variant), confirming that the valine side chain was not critical and that a smaller side chain at this position is, in fact, marginally more favorable. Finally, the P9Aib substitution conferred a 2-fold improvement in affinity, which may result from slightly more favorable side chain orientations of the critical neighboring Arg8 and Asp10 residues.

Overall, the data generated using the two panels of variants indicated that improvements in affinity for Cdc42 in P7 were possible using targeted amino acid substitutions and that it was also possible to incorporate non-natural amino acids without disrupting binding.

### Incorporation of a Modified Disulfide into the Cyclic Peptides

With the aim of retaining the bioactive conformation of the library-derived disulfide bond but incorporating a modified link with potentially enhanced stability, further variants were designed with homocysteine substitutions at positions 4 (P7 C4hC) and 13 (P7 C13hC). These allow disulfide contraction to a thioether, maintaining a similar cyclization linkage length and with fewer possible stereoisomer products. Our previously published structure was of P7 C4hC, i.e. incorporating a homocysteine (hCys, hC) at position 4.^19^ The disulfide linked homocysteine variants were tested for their affinity for Cdc42 by competition SPA (Figure 4A, Table 3). The substitution at residue 13 resulted in no change in binding affinity, while the inclusion of a homocysteine residue at position 4 yielded a modest 2-fold increase in affinity for Cdc42, albeit within experimental errors.

**Table 3:** Affinities of Homocysteine Containing P7 Variants for Cdc42

**Variant**	**Sequence**	***K***_**d**_ ^a^ **(nM)**
**P7**	PSICHVHRPDWPCWYR	57 ± 26
**P7 C4hC**	PSI**hC**HVHRPDWPCWYR	28 ± 9
**P7 C13hC**	PSICHVHRPDWP**hC**WYR	61 ± 37

a Affinities calculated from competition SPAs against a Cdc42-ACK complex. Average *K*_d_ values are quoted with errors from curve fitting.

**Figure 4:**
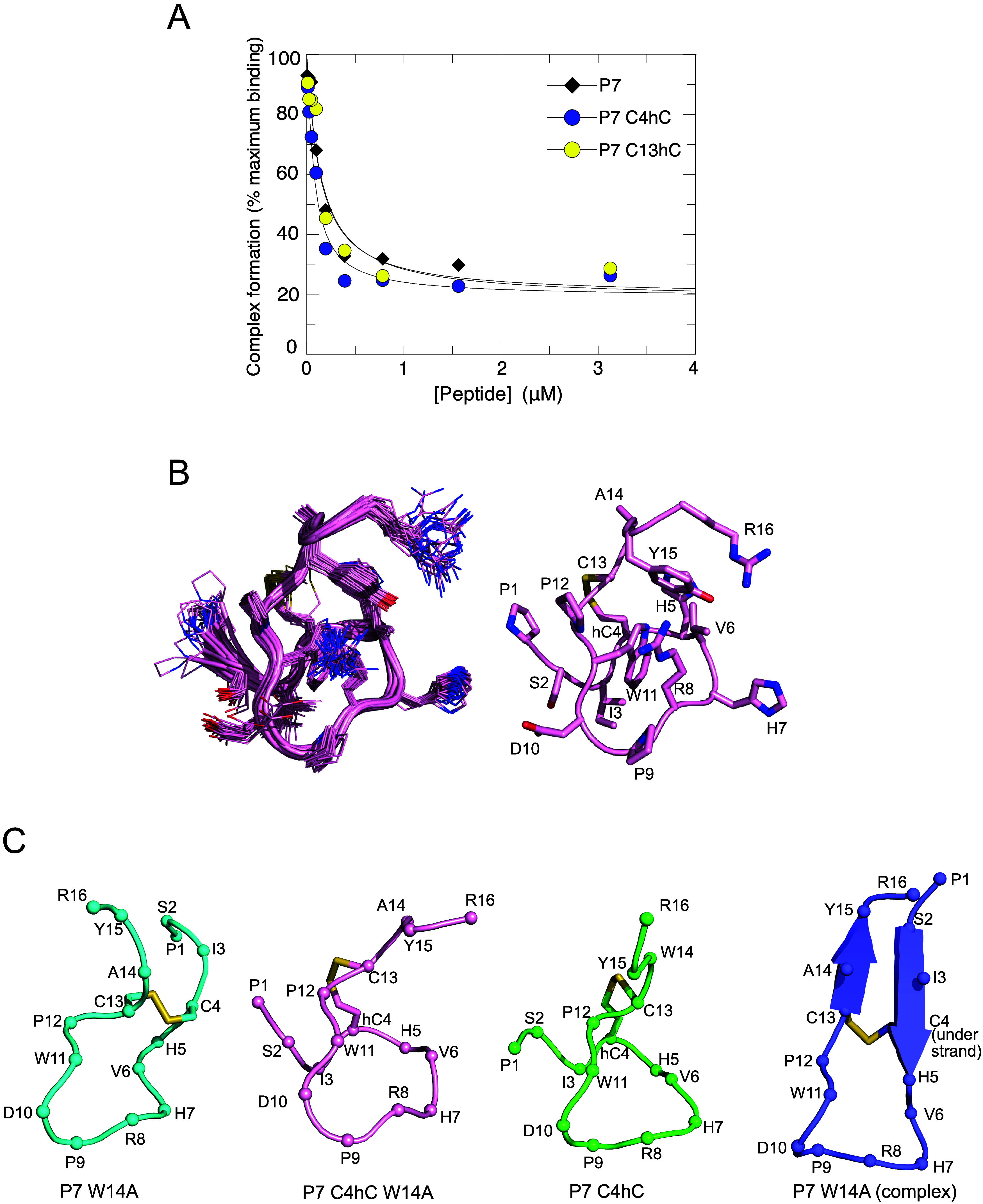
Homocysteine substituted peptides. A. Affinity of homocysteine substituted P7 for Cdc42. Displacement of [^3^H]­GTP·Cdc42 from GST-ACK by P7 variants. Increasing concentrations of each peptide were titrated into 30 mM [3H]­GTP·Cdc42 and GST-ACK 448–489. The *K*_d_ value for Cdc42 binding to GST-ACK 448–489 was fixed to the value (30 nM) obtained in direct SPAs. The data were fitted to an isotherm describing a full competition model to calculate the *K*_d_ values.^33^ The data and curve fits are displayed as a percentage of the maximal SPA signal, shown combined for repeats and the numerical *K*_d_ values and associated errors are presented in Table 3. B. Structure of P7 C4hC,W14A peptide. The family of structures is shown on the left and the closest structure to the mean is on the right. The backbone is shown as a cartoon and the side chains are colored as follows: carbon pink, oxygen red, nitrogen blue, sulfur yellow. C. Comparison of P7 W14A (cyan, PDB 9grk), P7 C4hC W14A (pink, PDB 9grl), P7 C4hC (green, PDB 6r28) peptide structures and P7 W14A from the Cdc42 complex (purple, PDB 9grm). Each structure is shown as a cartoon with the Cα atoms represented as spheres and labeled with the residue number. The disulfide bond is shown in yellow in each structure. The structures were aligned so that the apex of the loop, comprising residues 7–10, is in a similar orientation.

To understand the consequences of the hCys4 substitution we determined the structure of the W14A peptide with a hCys at position 4 (P7 C4hC,W14A) (Figure 4B). Like the free P7 W14A peptide, P7 C4hC,W14A has no fixed secondary structure and the disulfide bond constrains a loop with Arg8 and Asp10 at the tip. Comparison of the structures of P7 W14A and P7 C4hC,W14A (Figure 4C) reveals, however, that the orientation of the N- and C-termini relative to the loop are very different. The structure of P7 C4hC (PDB 6r28) shows that this change in orientation is dependent on the presence of the hCys, since P7 C4hC, and P7 C4hC,W14A have similar structures (Figure 4C). In the hCys-Cys peptides, the ends cross over, so that the N-and C-termini are on the opposite sides with respect to the loop, compared to P7 W14A. The orientation of the peptide termini in the P7 W14A peptide is, however, more like the final orientation of the peptide in the complex with Cdc42 (Figure 4C). Assuming that P7 C4hC,W14A binds Cdc42 in a similar orientation to P7 W14A, it is difficult to rationalize the similar affinities of P7 and P7 C4hC based on the free peptide structures. It is possible that the extra methylene group in hCys4 allows more flexibility around Ile3 and His5, both of which are buried in the interface. This would favor induced fit of these side chains, allowing them to make close contacts with Cdc42 despite the free peptide sampling a different conformation.

### Alanine Scanning of P7 C4hC

As the P7 C4hC substitution retained similar, or even modestly enhanced (Table 3), affinity for Cdc42 but had the potential to facilitate a modified cyclizing linkage, we employed alanine scanning in the context of the P7 C4hC peptide, to analyze side chain contributions to Cdc42 binding in this context (Figure 5, Table 4). The results indicated that most alanine substitutions were detrimental to affinity. Six substitutions were neutral: P1A, V6A, P9A, P12A, W14A and Y15A. Although alanine substitutions at Pro1, Val6 and Pro9 decreased the affinity, it was by less than 2-fold and therefore these represented positions potentially tolerable to mutation. Interestingly, three of the four structural variants tested in P7 that enhanced affinity were also modifications introduced at these positions (Figure 3). It was particularly noteworthy that the effects of the alanine substitutions on affinity for Cdc42 in P7 and P7 C4hC were significantly different (Tables 1 and 4).

**Figure 5:**
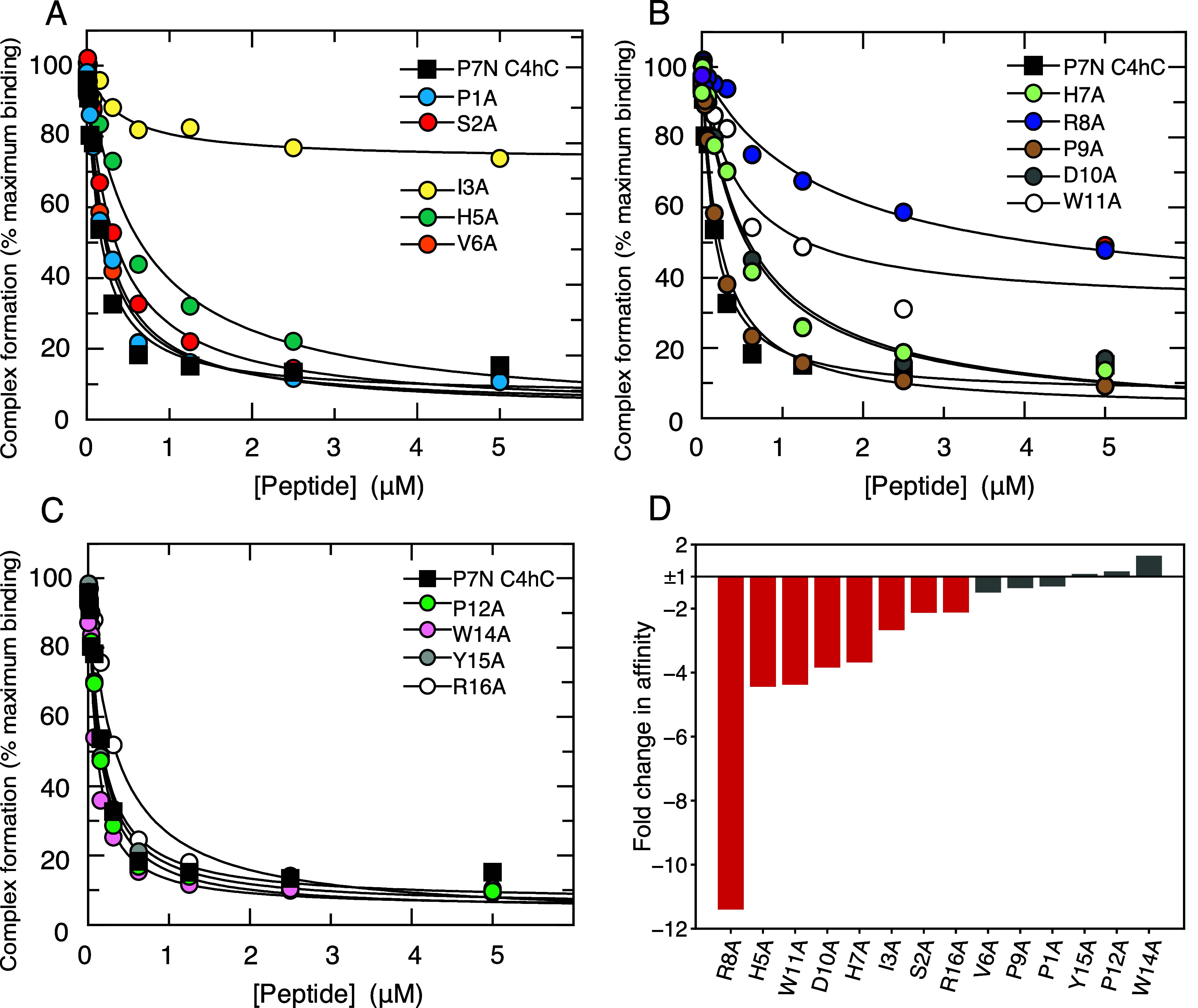
Affinity of P7 C4hC Alanine Variants for Cdc42. A–C. Displacement of [^3^H]­GTP·Cdc42 from GST-PAK1 by P7 C4hC variants. Increasing concentrations of each peptide were titrated into 20 mM [3H]­GTP·Cdc42 and GST-PAK1 75–132. The *K*_d_ value for Cdc42 binding to GST-PAK1 75–132 was fixed to the value (20 nM) obtained in direct SPAs. The data were fitted to an isotherm describing a full competition model to calculate the *K*_d_ values.^33^ The data and curve fits are displayed as a percentage of the maximal SPA signal, shown combined for repeats and the numerical *K*_d_ values and associated errors are presented in Table 4. D. Bar chart displaying the average fold change of each alanine variant relative to the control. Gray bars represent variants that bound with an affinity within ± 2-fold of the control (which were considered not significant) and red bars represent variants that bound with at least 2-fold lower affinity than the control.

**Table 4:** Affinities of Alanine Scan P7 C4hC Variants for Cdc42

**Variant**	**Sequence**	**Average *K***_**d**_ ^a^ **(nM)**	**Δ*G*** **(cal/mol)**	**ΔΔ*G*** **(cal/mol)**	**Fold Change in affinity**
**P7 C4hC**	PSIhCHVHRPDWPCWYR	61 ± 22	–9668		
**P1A**	**A**SIhCHVHRPDWPCWYR	80 ± 13	–9512	156	1.3 ↓
**S2A**	P**A**IhCHVHRPDWPCWYR	131 ± 26	–9227	440	2.1 ↓
**I3A**	PS**A**hCHVHRPDWPCWYR	164 ± 111	–9096	572	2.7 ↓
**H5A**	PSIhC**A**VHRPDWPCWYR	266 ± 62	–8814	853	4.4 ↓
**V6A**	PSIhCH**A**HRPDWPCWYR	92 ± 23	–9430	237	1.5 ↓
**H7A**	PSIhCHV**A**RPDWPCWYR	226 ± 59	–8910	758	3.7 ↓
**R8A**	PSIhCHVH**A**PDWPCWYR	696 ± 254	–8252	1416	11.4 ↓
**P9A**	PSIhCHVHR**A**DWPCWYR	83 ± 14	–9491	177	1.4 ↓
**D10A**	PSIhCHVHRP**A**WPCWYR	236 ± 58	–8884	784	3.9 ↓
**W11A**	PSIhCHVHRPD**A**PCWYR	269 ± 125	–8807	860	4.4 ↓
**P12A**	PSIhCHVHRPDW**A**CWYR	53 ± 10	–9756	–88	1.2 ↑
**W14A**	PSIhCHVHRPDWPC**A**YR	37 ± 11	–9958	–290	1.6 ↑
**Y15A**	PSIhCHVHRPDWPCW**A**R	57 ± 11	–9715	–47	1.1 ↑
**R16A**	PSIhCHVHRPDWPCWY**A**	130 ± 36	–9230	438	2.1 ↓

a Affinities calculated from competition SPAs against a Cdc42-PAK complex. Average *K*_d_ values are quoted with errors from curve fitting.

Overall, the P7 C4hC peptide is more tolerant to alanine substitutions: this is particularly evident for P12A, which had the biggest impact on P7 but did not affect P7 C4hC. This supports the hypothesis that the effect of the P12A substitution in P7 was structural, so that in the presence of hCys the flexibility afforded by the extra methylene allows binding. The proline at position 12 forms a kink in the backbone of P7 W14A in the complex, pushing the peptide backbone toward the neighboring strand and allowing the disulfide bond to form. In the P7 C4hC version of the peptide, the extra methylene group means that Cys13 can be slightly further away and still form the disulfide bond, hence P12A is tolerated. Interestingly, I3A, H5A, H7A and W11A although still deleterious, have smaller effects, suggesting that the C4hC of P7 C4hC can compensate for some of the loss in interactions. R8A has the biggest effect in the context of P7 C4hC, exhibiting a similar loss of affinity as it did in P7, indicating that its hydrogen bond is essential for binding. Finally, W14A, which increased the binding in P7 had a smaller effect in P7 C4hC. Presumably this is because Trp14 can move further away from Cdc42 in the presence of hCys and hence makes fewer unfavorable interactions.

## Conclusions

To date, peptide design has been commonly applied to engineering stabilized alpha helical conformations,^5,16,42^ but is less frequently employed to exploit β-sheet structures. The P7 W14A peptide inhibitor identified in this work is proof-of-principle that selective targeting of the antiparallel β2-strand of a small GTPase can be achieved. Although the extended β-sheet formed by P7 W14A is offset compared to that adopted by the CRIB effectors, it still competes with their binding. The position of the peptide interface also means that the switch regions of the G protein are not involved in the interaction, thus rendering the complex nucleotide independent. Such a mode of action could have relevance to targeting GTPases, where selectivity for both nucleotide forms has therapeutic relevance.

Structural details of the β-hairpin interface, open design opportunities for more selective binders at this artificially formed PPI. We have already identified variants that improve binding (Figure 3), indicating that more binding capacity is available in this scaffold. Stabilizing the interactions of the β-hairpin formed by P7 W14A with Cdc42 could be further enhanced through N-amination,^43^ the incorporation of β-mimetic residues,^44^ β-peptide foldamers,^45^ or covalent restraints, which could also improve proteolytic stability and cellular permeability.^46,47^ Predicting the effects of substitution on the overall performance of new variants is complicated by the conformational selection seen on target engagement. Changes that restrict the conformational flexibility of the free peptide to minimize the entropic penalty of adopting the hairpin structure will have to be balanced with maintaining favorable interactions with the target. The introduction of non-natural amino acids (2-aminoisobutyric acid or 5-bromotryptophan) was not only well-tolerated by the peptide inhibitors in this study but also increased the affinity for their target, Cdc42. These are important observations for the development of these peptides as lead therapeutics, as some non-natural amino acids are resistant to protease degradation and their presence could improve delivery and intracellular stability. Introduction of a homocysteine in P7 C4hC,W14A was also well tolerated and could similarly have utility in enhancing stability of the peptide. Disulfide cyclized peptides are not generally considered stable under intracellular conditions. We have found that the disulfide bonded conformation of these peptides is crucial to their activity (our unpublished results), so this would have to be precisely mimicked in a linkage with increased biostability. Contraction of the disulfide to a lanthionine bond produced multiple products but only one was biologically active. Introduction of a homocysteine reduces the number of possible products making synthesis and purification more viable.

We also predict that the combination of favorable replacement residues identified here could produce a peptide inhibitor with subnanomolar affinity for Cdc42 and that a strategy based on this peptide scaffold could yield specific inhibitors of other Ras GTPases. These targets have important roles in cell motility, and therefore in cancer progression and metastasis. Intracellular delivery of these inhibitors could be achieved by addition of a CPP to achieve a therapeutic effect. Indeed, we previously showed that an R9 CPP fused to the P7 peptide facilitated entry to a K-Ras mutant cancer cell line and the fused peptide mediated antimigratory and antiproliferative effects.^19^ The fusion of a CPP with an inhibitory peptide is an instinctive progression for peptide biologics and has been successfully employed to modulate multiple protein–protein interaction targets in cancer cells.^48,49^ Promising new strategies for the use of fused CPPs, specifically for anticancer treatments, have been emerging and several CPP-based therapies have entered clinical trials, although none are yet approved.^50^ Alternatively, other delivery vehicles could also be exploited e.g. nanoparticles.

Overall, the P7 W14A peptide inhibitor represents a potential candidate as a standalone inhibitor or as a basis for the design of proximity-induced therapeutics, including proteolysis-targeting chimeras (PROTACs). This class of constrained β-hairpin peptides therefore provides a platform to both develop experimental probes and for the therapeutic inhibition of Cdc42. Moving forward, these scaffolds also have potential applicability for targeting other Ras superfamily GTPases.

## Supplementary Material



## Data Availability

All other data are contained within the manuscript and Supporting Information. Structures reported in this work have been deposited with the PDB, with accession codes 9grk, 9grl and 9grm.

## Notes

Table S1: Summary of NMR Data for Peptides. Table S2: Experimental restraints and Structural Statistics for the Cdc42-W14A complex. Figure S1: ^15^N HSQC experiments of free P7 W14A and in a complex with 2 mol equiv of unlabeled Cdc42·GMPPNP. Figure S2: NOEs observed between the first β-strand of P7 W14A and β2 of Cdc42. Figure S3: NOEs observed between Cdc42 helix α5 and P7 W14A peptide. Figure S4: Overlay of P7 W14A binding to Cdc42 with the CRIB effectors ACK, WASP and PAK4 Figure S5: Sequence alignments of small G proteins tested for binding to peptide P7. (PDF)

## Notes

Cdc42: UniProtKB P60953 WASP: UniProtKB P42768 PAK1: UniProtKB Q13153 ACK: UniProtKB Q07912

## 2

Deliver Biosciences, The London Bioscience Innovation Centre, 2 Royal College Street, London NW1 0NH, U.K
