# Supplementary material for: Cyclized Peptide Inhibitors of the Small G Protein Cdc42 Mimic Binding of Effector Proteins
Source: Biochemistry. 2026 Jan 21;65(3):297–310. doi: 10.1021/acs.biochem.5c00616 (PMC12874369; doi:10.1021/acs.biochem.5c00616)
Supplement: Supplementary file 1 [file bi5c00616_si_001.pdf]

Supporting Information

Cyclized peptide inhibitors of the small G protein Cdc42 mimic binding of effector proteins

Natasha P. Murphy[1], George J.N. Tetley[1,2], Jefferson Revell[3], Helen R. Mott[1*] and Darerca Owen[1*]

[1]Department of Biochemistry, University of Cambridge, 80 Tennis Court Road, Cambridge. CB2 1GA. U.K.

[2]Present address: Deliver Biosciences, The London Bioscience Innovation Centre, 2 Royal College Street, London. NW1 0NH

[3]AstraZeneca, BioPharmaceutical R&D, Discovery Sciences, Cambridge Biomedical Campus, 1 Francis Crick Ave, Cambridge, CB2 0AA, U.K.

Table S1: Summary of NMR Data for Peptides.

Table S2: Experimental restraints and Structural Statistics for the Cdc42-W14A complex.

Figure S1: 15N HSQC experiments of free P7 W14A and in a complex with 2 molar equivalents of unlabelled Cdc42·GMPPNP.

Figure S2: NOEs observed between the first β-strand of P7 W14A and β2 of Cdc42.

Figure S3. NOEs observed between Cdc42 helix α5 and P7 W14A peptide.

Figure S4: Overlay of P7 W14A binding to Cdc42 with the CRIB effectors ACK, WASP and PAK4

Figure S5: Sequence alignments of small G proteins tested for binding to peptide P7.

Table S1: Summary of NMR Data for Peptides.

	P7 W14A<br>35 structures<br>pdb 9grk	P7N W14A<br>35 structures<br>pdb 9grl
Total NOEs	365	441
Unambiguous[a]	330	395
Ambiguous[a]	27	33
RMSD (Å) bb	0.61 (0.11) [b]	0.40 (0.15)
RMSD (Å) heavy	1.11 (0.20)	0.75 (0.19)
**RMS deviations**		
From NOE restraints (Å)	0.023 (0.0016)	0.028 (0.0040)
From idealised geometry:		
Bonds (Å)	0.0047 (0.00021)	0.0047 (0.00047)
Angles (˚)	0.92 (0.030)	0.83 (0.043)
Impropers (˚)	1.5 (0.19)	1.4 (0.25)

[a] at the end of the 8th iteration

[b] Average shown. Numbers in brackets are the standard deviation

Table S2: Experimental restraints and Structural Statistics for the Cdc42-W14A complex[a].

Experimental restraints

Distance restraints:			
Total non-degenerate	3868		
Unambiguous	3302		
Ambiguous	556		
Unambiguous intermolecular	99		
Dihedral angle restraints	324		
Hydrogen bond restraints	100		
		**<SA> [b]**	**<SA>$_c$ [c]**
**Coordinate precision for well-ordered regions**			
RMSD of all backbone atoms (Å)[d]		0.74 ± 0.14	0.57
RMSD of all heavy atoms (Å)[d]		1.26 ± 0.16	1.09
RMSD of backbone atoms without switch regions (Å)[e]		0.62 ± 0.09	0.45
RMSD of all heavy atoms without switch regions (Å)[e]		1.10 ± 0.11	0.90
**Ramachandran analysis for all residues**			
Residues in most favoured regions:		86.5 %	90.0 %
Residues in additionally allowed regions:		12.1 %	9.4 %
Residues in disallowed regions:		1.4 %	0.6 %
**RMS deviations for all residues**			
from the experimental restraints:			
NOE distances (Å)		0.021 ± 0.0014	0.021
Dihedral angles (˚)		0.43 ± 0.063	0.41
from idealised geometry:			
Bonds (Å)		0.004 ± 0.0001	0.004
Angles (˚)		0.569 ± 0.020	0.55
Impropers (˚)		1.50 ± 0.01	1.35

[a] PDB accession code 9grm

[b] <SA> represents the average RMS deviations for the ensemble of 30 structures.

[c] <SA>$_c$ represents values for the structure that is closest to the mean.

[d] Calculated over Cdc42 residues 1-178 and all 16 residues of the peptide.

[e] Calculated over Cdc42 residues 1-28, 41-58, 71-178 and all 16 residues of the peptide.

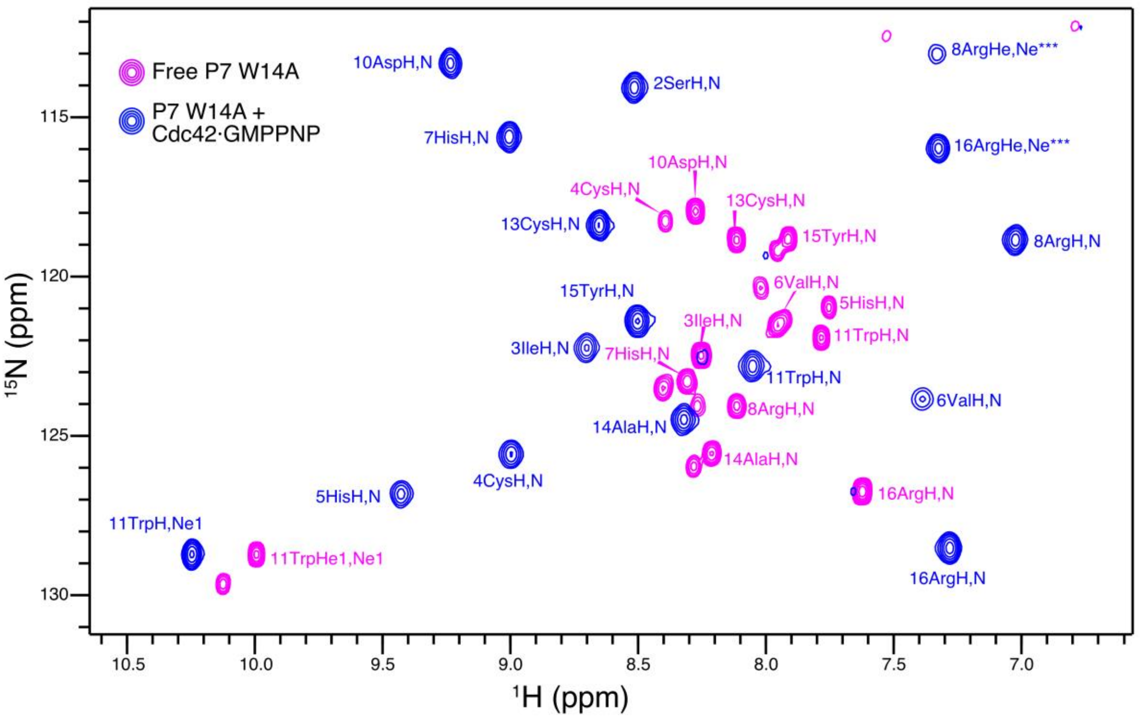

Figure S1: 15N HSQC experiments of free P7 W14A (pink) and in a complex with 2 molar equivalents of unlabelled Cdc42·GMPPNP (blue). Assignments of the major species are indicated. Assignments corresponding to aliased sidechain resonances are starred.

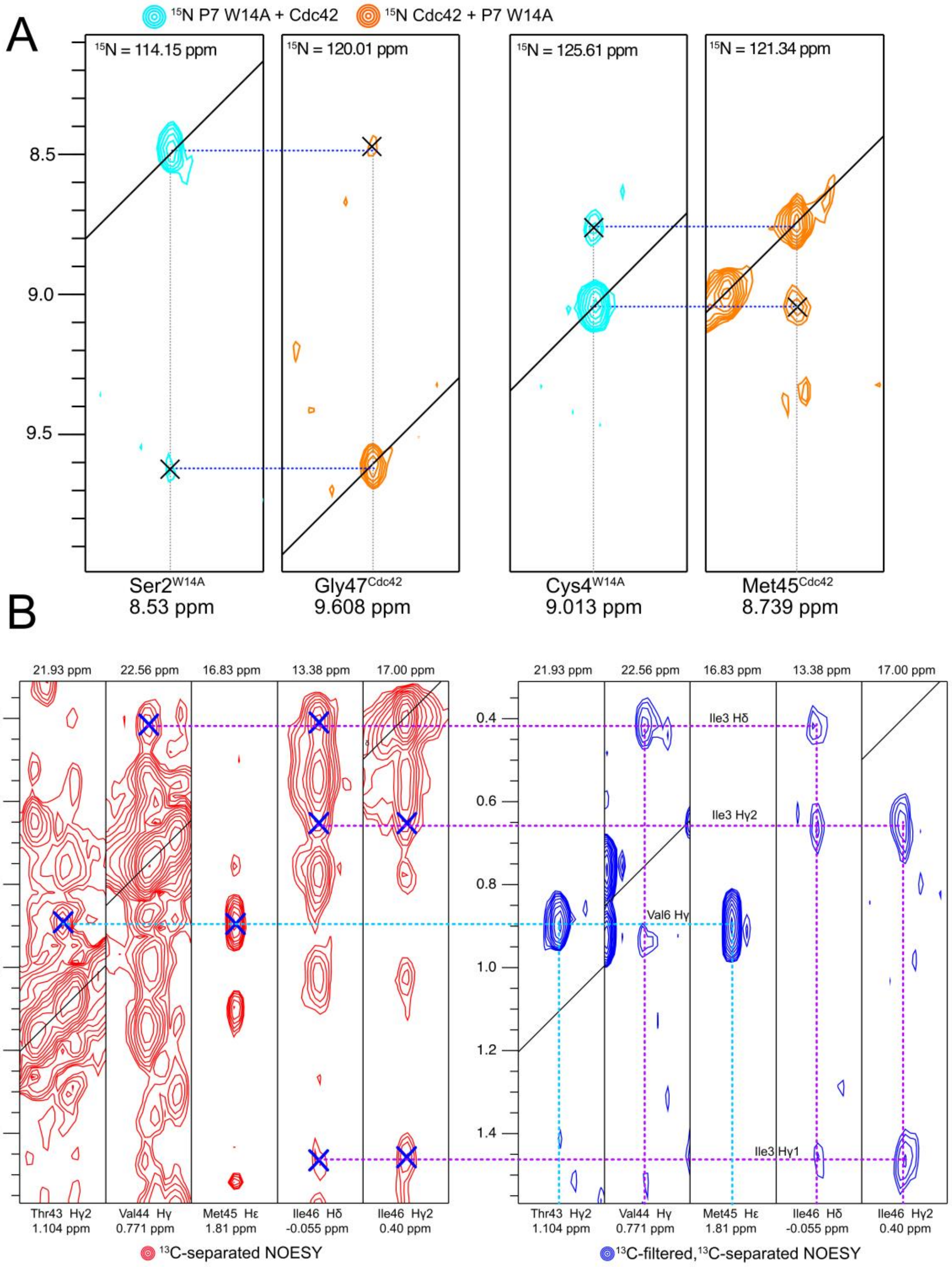

A
^{15}N P7 W14A + Cdc42
^{15}N Cdc42 + P7 W14A
^{15}N = 114.15 ppm
^{15}N = 120.01 ppm
^{15}N = 125.61 ppm
^{15}N = 121.34 ppm
8.5
9.0
9.5
Ser2^{W14A} 8.53 ppm
Gly47^{Cdc42} 9.608 ppm
Cys4^{W14A} 9.013 ppm
Met45^{Cdc42} 8.739 ppm
B
21.93 ppm
22.56 ppm
16.83 ppm
13.38 ppm
17.00 ppm
0.4
0.6
0.8
1.0
1.2
1.4
Ile3 Hδ
Ile3 Hγ2
Val6 Hγ
Ile3 Hγ1
Thr43 Hγ2 1.104 ppm
Val44 Hγ 0.771 ppm
Met45 Hε 1.81 ppm
Ile46 Hδ -0.055 ppm
Ile46 Hγ2 0.40 ppm
^{13}C-separated NOESY
^{13}C-filtered,^{13}C-separated NOESY

Figure S2: NOEs observed between the first β-strand of P7 W14A and β2 of Cdc42.

A. NH-NH NOEs in the ^{15}N-separated NOESY recorded on ^{15}N labelled peptide (cyan) or ^{15}N labelled Cdc42 (orange). Each strip is centred at a different NH position, with the ^{15}N chemical shift at the top and the ^{1}H chemical shift at the bottom. The diagonal is represented by a solid black line, intermolecular NH-NH NOEs are shown as dotted blue lines and cross peaks are marked with an X. B. Sidechain-sidechain NOEs in the ^{13}C-separated NOESY (red) and the ^{13}C-separated, ^{13}C-filtered NOESY (blue). Each strip is centred at a different methyl position, with the ^{13}C chemical shift at the top and the ^{1}H chemical shift at the bottom. The diagonal is represented by a solid black line. Positions of X-filter peaks in the ^{13}C-separated NOESY are marked with a blue X. Intermolecular NOEs between Val44/Ile46 (on one side of the β-sheet) and Ile3 are shown as magenta dotted lines. NOEs between Thr43/Met45 (on the other side of the β-sheet) and Val6 are shown as cyan dotted lines.

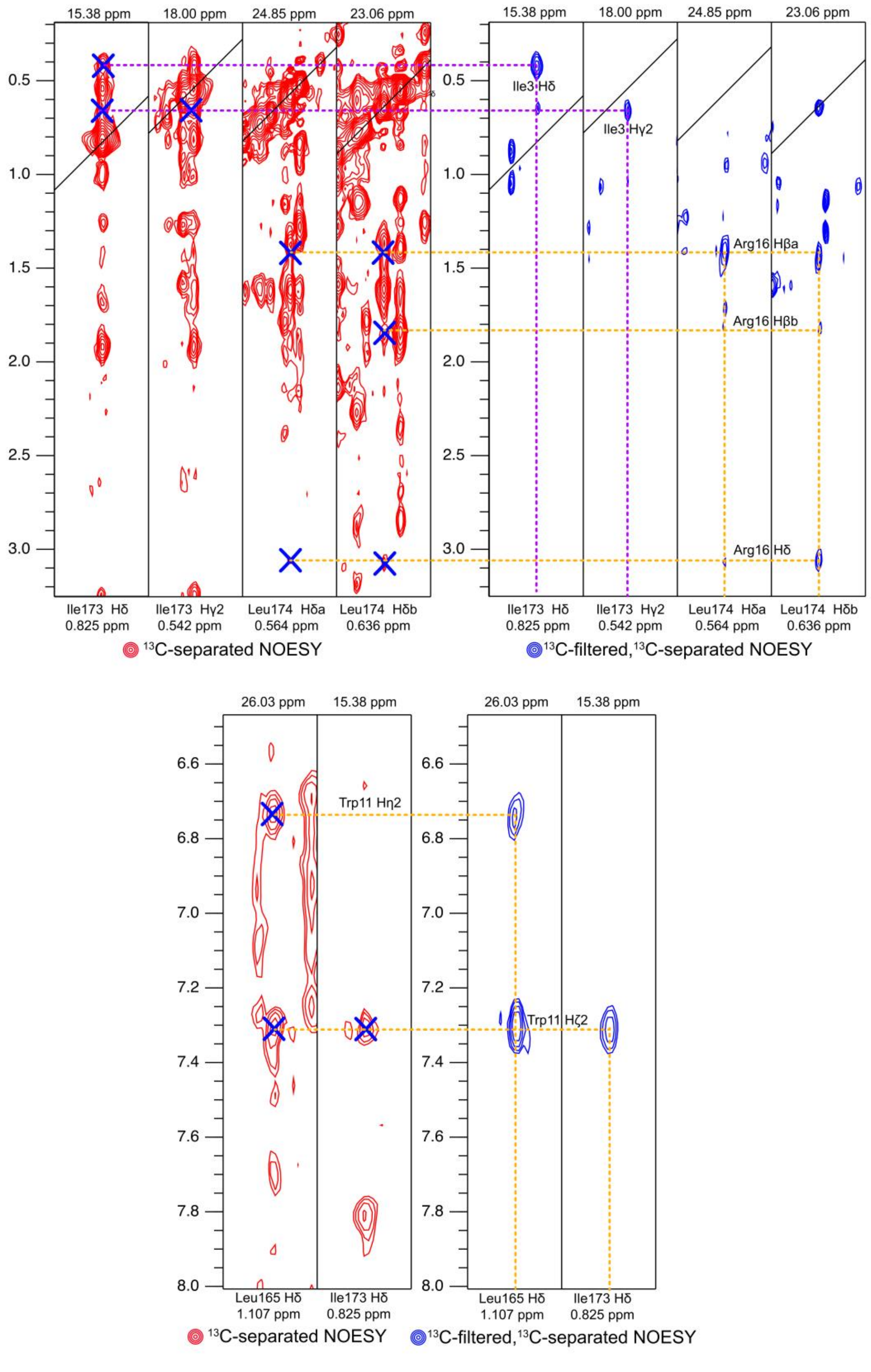
15.38 ppm
18.00 ppm
24.85 ppm
23.06 ppm
Ile3 Hδ
Ile3 Hγ2
Arg16 Hβa
Arg16 Hβb
Arg16 Hδ
Ile173 Hδ 0.825 ppm
Ile173 Hγ2 0.542 ppm
Leu174 Hδa 0.564 ppm
Leu174 Hδb 0.636 ppm
¹³C-separated NOESY
¹³C-filtered,¹³C-separated NOESY
26.03 ppm
15.38 ppm
Trp11 Hη2
Trp11 Hζ2
Leu165 Hδ 1.107 ppm
Ile173 Hδ 0.825 ppm
¹³C-separated NOESY
¹³C-filtered,¹³C-separated NOESY

Figure S3: NOEs observed between Cdc42 helix α5 and P7 W14A peptide. Sidechain-sidechain NOEs in the ^{13}C-separated NOESY (red) and the ^{13}C-separated, ^{13}C-filtered NOESY (blue) are shown. Each strip is centred at a different methyl position, with the ^{13}C chemical shift at the top and the ^{1}H chemical shift at the bottom. The diagonal is represented by a solid black line. Positions of X-filter peaks in the ^{13}C-separated NOESY are marked with a blue X. Intermolecular NOEs involving P7 W14A Ile3 in the first β-sheet are shown as magenta dotted lines. NOEs involving Trp11 and Arg16 in the C-terminal part of the peptide are shown as orange dotted lines.

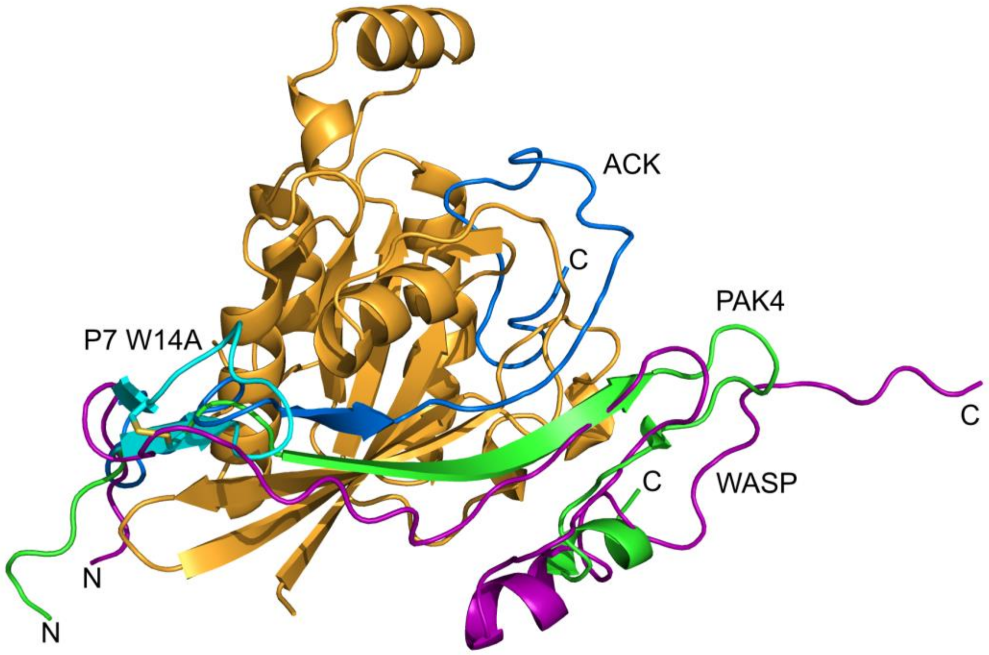

Figure S4: Overlay of P7 W14A bound to Cdc42 with the CRIB effectors ACK, WASP and PAK4. The four structures were overlaid over Cdc42. Cdc42 in the P7 W14A complex is shown in orange, P7 W14A is cyan, ACK is blue, WASP is magenta and PAK4 is green.

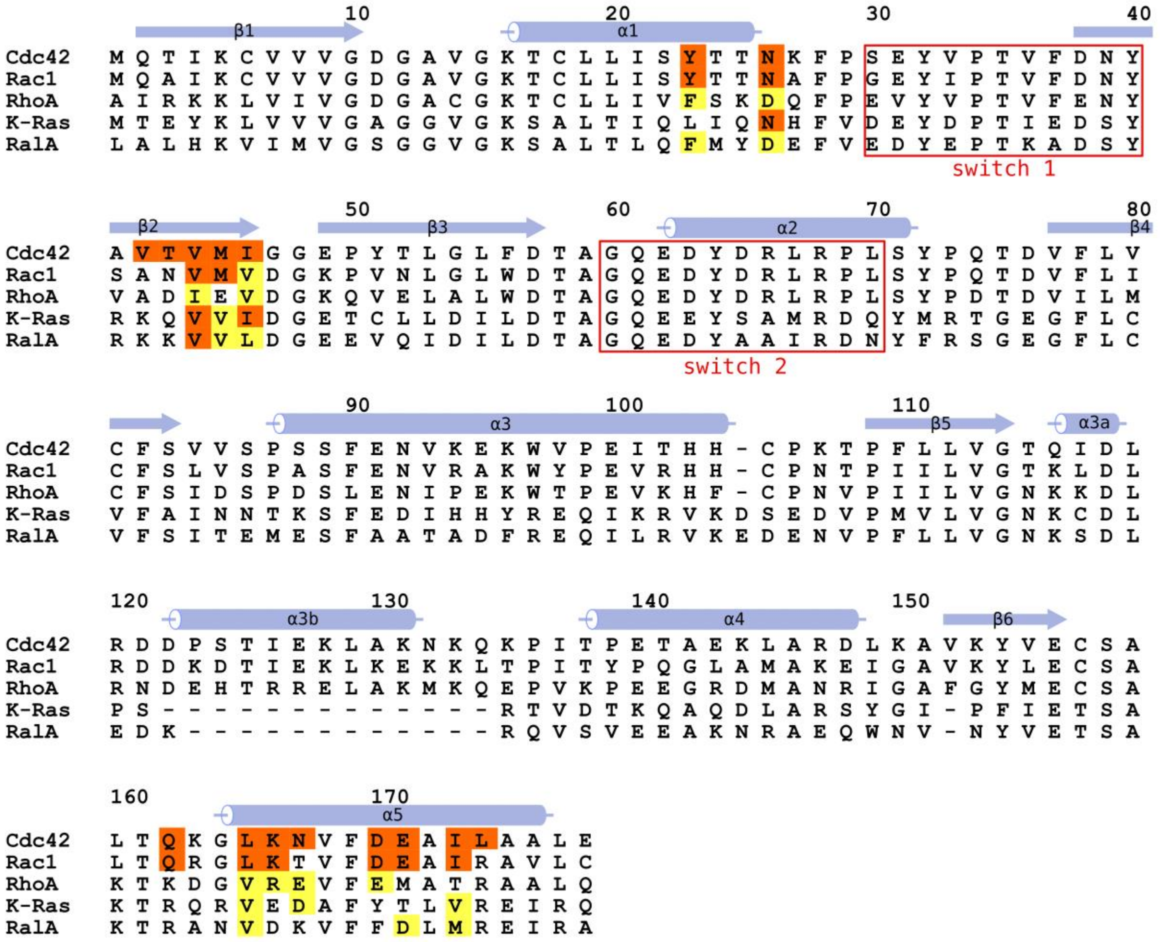

Figure S5: Sequence alignments of small G proteins tested for binding to peptide P7. Cdc42 residues that interact with P7 W14A are shaded orange. Residues conserved in the other 4 proteins are shaded orange (identical) and yellow (homologous). The secondary structure of Cdc42 is shown above the sequence as arrows (β-strands) and cylinders (α-helix). Numbers above the sequence are Cdc42 residue numbers.
